# Supplementary material for: End Sequence Analysis Toolkit (ESAT) expands the extractable information from single-cell RNA-seq data
Source: Genome Res. 2016 Oct;26(10):1397–410. doi: 10.1101/gr.207902.116 (PMC5052061; doi:10.1101/gr.207902.116)
Supplement: Supplemental Material [file supp_gr.207902.116_Supp_Materials.tar.gz › ESAT-esat_v0.1/doc/html/functions_vars_r.html]

ESAT: Class Members - Variables


ESAT


- Main Page
- Related Pages
- Packages
- Classes
- Files

- Class List
- Class Index
- Class Hierarchy
- Class Members

- All
- Functions
- Variables
- Enumerator

- a
- b
- c
- d
- e
- f
- g
- h
- i
- k
- l
- m
- n
- o
- p
- q
- r
- s
- t
- u
- v
- w


All Classes Namespaces Files Functions Variables Enumerator Pages

### - r -

- random
  : broad.pda.seq.protection.MultiSampleScanPeakCaller
- RANDOM\_SEED
  : broad.pda.seq.rap.BuildRatioNullDistribution
  , broad.pda.seq.rap.ShuffleBAM
- READ\_LIST\_FILE
  : broad.pda.samtools.FilterFastqReads
- readCigarFlag
  : umms.core.writers.PairedEndWriter
- readStartFlag
  : umms.core.writers.PairedEndWriter
- REF
  : broad.pda.seq.rap.DistanceToBED
- REFERENCE\_SEQUENCE
  : broad.pda.seq.rap.GCContent
- REG\_PATTERN
  : broad.core.annotation.BasicGenomicAnnotationReader
- REGION
  : broad.pda.seq.rap.GenomeCommandLineProgram
- region
  : umms.core.coordinatesystem.ShuffledIterator< T extends Annotation >
- REMOVE\_MATE
  : broad.pda.samtools.FilterPairedReadsByMAQ
- removeAtEnd
  : broad.pda.samtools.TrimFastqReads
- removeAtStart
  : broad.pda.samtools.TrimFastqReads
- repeatMaskerAnnotationFactory
  : broad.core.annotation.AnnotationFactoryFactory
- RIGHT\_MARGIN
  : broad.core.annotation.BasicGenomicAnnotationReader
- RPKM\_OFFSET
  : umms.core.model.score.NewRatioScore
  , umms.core.model.score.RatioScore
  , umms.core.model.score.WindowAllScore
- RPKM\_RATIO\_OFFSET
  : broad.pda.seq.rap.SlideAndCalculate


---

Generated on Fri Aug 15 2014 15:11:17 for ESAT by  

 1.8.7
